# Incidence and prevalence of total joint replacements due to osteoarthritis in the elderly: risk factors and factors associated with late life prevalence in the AGES-Reykjavik Study

**DOI:** 10.1186/s12891-016-0864-7

**Published:** 2016-01-12

**Authors:** Helgi Jonsson, Sigurbjorg Olafsdottir, Solveig Sigurdardottir, Thor Aspelund, Gudny Eiriksdottir, Sigurdur Sigurdsson, Tamara B. Harris, Lenore Launer, Vilmundur Gudnason

**Affiliations:** Landspitalinn University Hospital, University of Iceland, Reykjavik, Iceland; University of Iceland, Reykjavik, Iceland; Icelandic Heart Association, Kopavogur, Iceland; National Institute on Aging, Bethesda, MD USA; Department of Rheumatology, Landspitalinn University Hospital, IS-108 Fossvogur, Reykjavik, Iceland

**Keywords:** Osteoarthritis, Arthroplasty, Epidemiology, Risk factors

## Abstract

**Background:**

Total joint replacements (TJRs) should be considered as one of few definite endpoints in osteoarthritis research. We analyzed factors associated with late-life prevalence and risk factors for incidence of TJRs due to osteoarthritis in a population based cohort.

**Methods:**

After exclusion of inflammatory arthritis and fractures as causes of TJR, 5170 participants in the AGES-Reykjavik Study (mean age (SD) 76.4(6), 58 % females) were included for osteoarthritis studies. Three thousand one hundred thirty-three of them had a follow-up visit 5 years later.

**Results:**

The prevalence of having at least one joint replacement operation due to OA was 13.6 % and the yearly incidence was 1.4 %/year during the five-year follow-up. Factors positively associated with late life prevalence of TJR included BMI, hand OA severity, female gender, finger length ratio and spine BMD. Risk factors for TJRs in the incidence group were symptoms at initial visit, prior TJR in the contralateral joint and BMI. Much stronger associations were seen for TKR than for THR with discriminatory analysis showing an AUC 0.71 for late life prevalence and 0.84 for the incidence.

**Conclusions:**

This study illustrates the importance of the different information expressed by late life prevalence vs. incidence on the factors associated with severe osteoarthritis of the knee and hip. The observation that prior TJR is a risk factor for subsequent TJR in the contralateral joint has not been described previously. The high power predictions for TKR suggest that a predictive model may be feasible, particularly if it can be extended by the addition of further predictive variables, perhaps through genetic, biomarker or imaging data.

## Background

Large joint osteoarthritis (OA) is a major cause of pain and disability worldwide. The disease process is heterogeneous with multiple joints affected and there is discordance between the anatomical state of the joint on imaging or clinical examination on one hand and symptoms and disability on the other [1, 2]. The causes of OA are largely unknown, but a number of genetic and environmental causes have been implicated. Local causes such as trauma, malalignment, dysplasia and hypermobility may lead to OA in the involved joints [3–5]. Body mass index (BMI) and heavy work is also associated with knee and hip OA [6, 7], but there is also evidence of systemic factors with polyarticular involvement, particularly associated with hand OA [8].

Compared with other chronic diseases there is a lack of definite measures in OA that can be useful as endpoints in intervention studies [9]. Total joint replacement operations of the knee and hip (TJRs) are the treatment of choice in the most severe forms of knee and hip OA. Thus, TJRs are a surrogate marker of severe OA and a possible endpoint, but unfortunately, there are intrinsic problems associated with this endpoint such as access barriers due to geographical and financial variations in the availability of the resources for TJR and also in the willingness of patients to have such operations [10–12].

There have been a number of studies addressing factors associated with the risk of having TJRs [13, 14], and recently, an attempt was made to create a predictive model for the incidence of knee osteoarthritis [15]. A predictive model identifying those likely to need total joint replacements for OA would be a very valuable tool with regard to intervention measures in large joint OA and the planning of the resources to meet that need.

Our group has previously described positive associations between the prevalence of total knee replacements (TKRs) and BMI, hand osteoarthritis severity, total hip replacements (THRs), and finger length ratios [16, 17]. Education, occupation and physical exercise however, showed only minor associations with TJR [18] in the ongoing Age, Gene\Environment Susceptibility-Reykjavik Study (AGES-Reykjavik Study), a population-based cohort study of older Icelanders [19]. In Iceland the health care system is socialized with supposedly equal access for all.

The objective of the present study was twofold, to study factors associated with late life point prevalence of TJRs of the knee and hip and secondly to analyze risk factors associated with TJRs in the prospective 5-year follow-up. It is our hope that this information may prove to be a step towards a predictive model for the need for TJRs.

## Methods

The AGES-Reykjavik Study is a prospective study of 5764 men and women, age 66–96 years, based on a representative sample of the population of Reykjavik, Iceland.

Participation was 71.8 %. Informed consent was obtained from all participants [19]. During 2007–2011, all living participants were invited to a five year follow-up visit and the participation was 71 %.

Information about hip and knee joint replacements was obtained by computerized tomography (CT) anterior scout scans. After excluding participants with inflammatory arthritis and joint replacements due to fractures using a nationwide register of fractures [20], 5170 participants (2195 males, 2975 females, mean age 76.4(5.5)) were eligible for osteoarthritis studies [14]. The incidence group constituted the 3133 subjects who attended the follow-up visit approximately 5 years later (AGES-Reykjavik II) and had a second CT scan (1312 males, 1821 females, mean age 79.9(4.8)).

Information about TJR’s was obtained at both first visit and at follow-up, but all other data was obtained at first visit. The prevalence reported included all TJRs at the end of AGES II. Incident joint replacements due to fractures were not included. Joint replacements were registered on a patient level by site as present and absent in the prevalence analysis and as new joint replacements present or absent in the incidence analysis. No revisions on previous TJR’s were included in the incidence reports.

Quantitative CT (QCT) scans for measurement of BMD in the lumbar spine were obtained with a CT Sensation 4 detector scanner (Siemens Medical Systems, Erlangen, Germany) as described previously [21].

To calibrate CT Hounsfield units to equivalent bone mineral concentration, all subjects were positioned supine on a calibration phantom (Image Analysis, Columbia, KY, USA) containing calibration cells of 0.75 and 150 mg/cm^3^ equivalent concentrations of calcium hydroxyapatite. The lumbar spine included a spiral scan of the L1 and L2 vertebras (1-mm slice thickness, pitch = 1.120 kilovolt peak [kVp], 150 milliamp seconds [mAs]). QCT images were transferred to a network of computer workstations and processed to extract measures of bone mineral density (vBMD) and bone size using analysis techniques described previously [22]. For each trabecular, cortical, and integral region of interest, vBMD (grams per cubic centimetre), bone mineral content (grams), and bone volume (cubic centimetres) were computed. Spine trabecular bone mineral density (BMD) was calculated from an elliptical region in the anterior midvertebra. Integral volume BMD (vBMD) was computed in grams per cubic centimetre) for a region encompassing the entire midvertebra excluding transverse elements. Every 3 months throughout the study the interobserver variability for the whole group of observers were assessed. The average interobserver variability for the average integral vBMD of L1 and L2 excluding the transverse elements were 3.0 % (coefficient of variation) and 0,98 (Spearman’s correlation). The average integral vBMD of L1 and L2 was used as an outcome variable and recorded as percent of average (100) in 5 age and gender categories.

Education was originally listed as one of 10 categories according to different schooling but later combined into 4 categories, in essence corresponding with: 1. Primary school, 2. Secondary school, 3. College education, 4. University education.

Other variables with previously reported positive associations with TJR included BMI, finger length ratio (1: fourth finger shorter than second finger, 2: second and fourth fingers equally long and 3: Fourth finger longer than second finger) and hand osteoarthritis severity (read from high quality photographs on a scale from 0 to 4 [17, 23]. Additional variables were smoking history (0 = never, 1 = former, 2 = current), High sensitivity C-reactive protein (Hs-CRP), Cholesterol, Triglycerides, Statin use, The methods for analysis of these variables have been described previously [8].

### Statistics

Logistic regression models were used to estimate the association of the prevalence and 5 year incidence of THR and TKR with selected predictor variables. The main variables were age, gender, BMI, finger length ratio, hand OA severity and BMD. Possible confounding variables adjusted for were education, smoking, Hs-CRP, cholesterol, triglycerides and use of statins. Two sets of models were run in the prevalence analysis, with and without adjustment for possible confounding variables. In the incidence analysis the history of previous hip and/or knee symptoms were added to the models and those who already had bilateral TKRs or THRs were excluded in the statistic calculations for the respective joint. The variables for finger length ratio and hand osteoarthritis severity were analysed on a linear scale to ease interpretation. Linearity of effects were inspected and significance of non-linear effects tested using orthogonal contrasts. Only linear effects were statistically significant. For each model the area under the receiver operating characteristic (ROC) curve was estimated along with the Nagelkerke R^2^. Results from models using finger length ratio and hand osteoarthritis severity as categorical variables vs. linear variables were compared to make sure estimates were similar. We used the SPSS 19 software package for the multivariable logistic analysis and R version 3.0 and the package pROC to estimate areas under the ROC curves [24].

### Ethical approval and informed consent

The study is approved by the Icelandic National Bioethics Committee, (VSN: 00-063) and the Data Protection Authority. All participants signed an informed consent declaration.

## Results

Five thousand one hundred seventy participants (2195 males, mean age 76.5(5.3), 2975 females, mean age 76.4(5.6)) were eligible for osteoarthritis studies [14]. Mean BMI for males was 26.9(3.8) and females 27.2(4.8).

The prevalence and incidence by gender and age-groups of participants having had TJR in the AGES-Reykjavik Study are shown in Table 1. The overall prevalence of those having at least one joint replacement operation due to OA was 13.6 % and the overall incidence in the follow-up group was 1.4 %/year during the five-year follow-up.

**Table 1:** Prevalence and five-year incidence of total joint replacements due to osteoarthritis

Prevalence at the end of AGES II (*n* = 5170)								
	Males				Females			
Age groups	n	TKR (%)	THR (%)	TJR (%)	n	TKR (%)	THR (%)	TJR (%)
−69	188	6.9	4.3	10.6	339	5.6	8.8	13
70–74	677	5.6	6.9	11.2	878	7.5	8.9	15.3
75–79	673	4.5	6.7	10.5	832	7.6	8.1	14.3
80–84	482	6.0	7.3	13.1	685	6.0	11.5	16.6
85+	175	4.3	8.6	11.4	241	4.6	12.9	17.0
Total (95%CI)	2195	5.2 (4.3–6.2)	6.8 (5.8–7.9)	11.4 (10.1–12.7)	2975	6.7 (5.8–7.6)	9.6 (8.5–10.6)	15.2 (13.9–16.5)
Incidence during five-year follow-up (*n* = 3133)^a^								
	Males				Females			
Age groups	n	TKR (%)	THR (%)	TJR (%)	n	TKR (%)	THR (%)	TJR (%)
−69	154	3.9	1.9	5.8	266	3.4	5.6	8.6
70–74	500	2.8	3.6	6.2	679	5.9	4.3	9.9
75–79	423	2.8	3.1	5.9	507	3.2	4.1	7.1
80–84	200	3.0	2.5	5.5	310	1.0	4.5	5.2
85+	35	0	2.9	2.9	59	1.7	1.7	3.4
Total (95%CI)	1312	2.9 (2.0–3.8)	3.0 (2.1–4.0)	5.9 (4.6–7.1)	1821	3.8 (2.9–4.7)	4.4 (3.5–5.3)	7.9 (6.7–9.1)

^a^The incidence calculations are limited to those completing the five-year follow-up

### Late-life TJR prevalence associations (Tables 2, 3 and 4)

**Table 2:** Study variables associated with the prevalence of total knee joint replacements

	TKR (*n* = 315)	*p* value	TKR	*p* value
	Multivariate 1		Multivariate 2	
Age (per year)	0.99 (0.97–1.01)	0.33	0.98 (0.96–1.01)	0.18
Female sex	1.20 (0.94–1.54)	0.15	1.21 (0.92–1.59)	0.18
BMI (per unit)	1.13 (1.10–1.16)	<0.0001	1,13 (1.10–1.16)	<0.0001
Finger length ratio (1–3)	1.40 (1.16–1.68)	<0.0001	1.40 (1.17–1.69)	<0.0001
Hand OA severity (0–4)	1.23 (1.13–1.34)	<0.0001	1.22 (1.12–1.33)	<0.0001
BMD (per 10 percent of predicted)	1.04 (1.00–1.08)	0.03	1,04 (1.01–1.08)	0.02
Education (1–4)			0.94 (0.82–1.08)	0.42
Smoking history (1–3)			0.90 (0.75–1.09)	0.28
hs-CRP			1.00 (0.98–1.02)	0.97
Cholesterol			0.93 (0.81–1.06)	0.27
Triglycerides			0.81 (0.66–1.01)	0.06
Statin use			0.96 (0.69–1.34)	0.82
*AUC (95 % CI):*	0.71 (0.67–0.73)		0.71 (0.67–0.73)	
*R* ^*2*^ *: Cragg-Uhler (Nagelkerke):*	0.08		0.08

**Table 3:** Study variables associated with the prevalence of total hip joint replacements

	THR (*n* = 435)	*p* value	THR	*p* value
	Multivariate 1		Multivariate 2	
Age (per year)	1.02 (1.00–1.04)	0.02	1.03 (1.01–1.04)	0.01
Female sex	1.42 (1.15–1.76)	0.001	1.52 (1.20–1.92)	<0.0001
BMI (per unit)	1.04 (1.01–1.06)	0.002	1.04 (1.01–1.06)	0.003
Finger length ratio (1–3)	1.05 (0.91–1.24)	0.52	1.05 (0.91–1.22)	0.49
Hand OA severity (0–4)	1.15 (1.07–1.24)	<0.0001	1.15 (1.07–1.24)	<0.0001
BMD (per 10 percent of predicted)	1.05 (1.02–1.08)	0.002	1.05 (1.02–1.08)	0.003
Education (1–4)			1.13 (1.01–1.26)	0.04
Smoking history (1–3)			1.04 (0.89–1.21)	0.67
hs-CRP			1.00 (0.99–1.02)	0.69
Cholesterol			0.99 (0.89–1.11)	0.89
Triglycerides			0.98 (0.83–1.45)	0.82
Statin use			1.10 (0.83–1.45)	0.53
*AUC (95 % CI):*	0.61 (0.59–0.64)		0.62 (0.59–0.64)	
*R* ^*2*^ *: Cragg-Uhler (Nagelkerke):*	0.03		0.03

**Table 4:** Study variables associated with the prevalence of total joint replacements (Knee or Hip)

	THR (*n* = 702)	*p* value	THR	*p* value
	Multivariate 1		Multivariate 2	
Age (per year)	1.01 (1.00–1.03)	0.11	1.01 (1.00–1.03)	0.15
Female sex	1.36 (1.15–1.62)	0.0005	1.41 (1.17–1.71)	<0.0001
BMI (per unit)	1.08 (1.06–1.1)	<0.0001	1.08 (1.06–1.1)	<0.0001
Finger length ratio (1–3)	1.22 (1.07–1.38)	0.002	1.22 (1.07–1.38)	0.002
Hand OA severity (0–4)	1.19 (1.13–1.27)	<0.0001	1.19 (1.12–1.26)	<0.0001
BMD (per 10 percent of predicted)	1.04 (1.02–1.07)	0.001	1.04 (1.02–1.07)	0.001
Education (1–4)			1.03 (0.94–1.13)	0.58
Smoking history (1–3)			0.95 (0.84–1.08)	0.46
hs-CRP			1.00 (0.99–1.02)	0.67
Cholesterol			0.95 (0.87–1.04)	0.31
Triglycerides			0.92 (0.80–1.06)	0.24
Statin use			1.04 (0.83–1.31)	0.75
*AUC (95 % CI):*	0.65 (0.63–0.67)		0.65 (0.63–0.67)	
*R* ^*2*^ *: Cragg-Uhler (Nagelkerke):*	0.06		0.03	

We did two separate multivariate analyses, initially using factors that had previously been implicated in the prevalence of OA. The second analysis also included a number of variables with possible associations. BMI showed the strongest positive association with TKR prevalence (OR 1.13(1.10–1.16), *p* = 1.8 × 10^−21^ per unit), and hand OA severity was also strongly associated (OR1.23(1.13–1.34), *p* = 2.7 × 10^−6^ per severity grade). There were also significant associations with finger length ratio, female gender and BMD but no associations were seen with, age, education, smoking history, Hs-CRP, Cholesterol, triglycerides or statin use. When all these factors were combined in one model, Area under curve (AUC) analysis revealed a discriminatory power of 0.71 (0.67–0.73).

Associations with THRs were much more modest with a total AUC of only 0.61 (0.58–0.64); the strongest positive association was with hand OA severity and significant associations were also seen for age, female gender, BMI and BMD as well as a weak positive association with education (OR 1.13(1.01–1.26), *p* = 0.04). Analysis of TJRs revealed no new associations, BMI and Hand OA severity showed the strongest associations but there were also significant associations with age, female gender, finger length ratio and BMD.

### TJR incidence risk factor analysis (Table 5)

**Table 5:** Risk factors for total joint replacements during a 5-year follow-up in the ages Reykjavik studies

	TKR (*n* = 105)^a^	*p* value	THR (*n* = 117)^b^	*p* value	TJR (*n* = 217)	*p* value
Age (per year)	0.96 (0.91–1.00)	0.07	0.97 (0.93–1.01)	0.14	0.96 (0,93–0,99)	0.01
Female sex	1.08 (0.68–1.74)	0.74	1.12 (0.73–1.72)	0.61	1.07 (0.78–1.48)	0.67
BMI (per unit)	1.13 (1.08–1.19)	<0.0001	1.01 (0.96–1.05)	0.81	1.07 (1.03–1.10)	0.0002
Finger length ratio (1–3)	1.47 (1.07–2,03)	0.02	1.04 (0.79–1.36)	0.78	1.22 (0.99–1.51)	0.06
Hand OA severity (0–4)	1.04 (0.89–1.22)	0.61	1.17 (1.02–1.34)	0.03	1.11 (1.00–1.24))	0.04
BMD (per 10 % of predicted)	1,03 (0,96–1,10)	0,4	0,99 (0,92–1,05)	0,67	1.00 (0,96–1,05)	0,94
Previous TKR	4.94 (2.42–7.71)	<0.0001	0.80 (0.37–1.72))	0.56	1.91 (1.16–3.14)	0.01
Previous THR	1.28 (0.68–2.40)	0.45	3.56 (2.04–6.22)	<0.0001	2.10 (1.30–3.39)	0.003
Knee symptoms^c^	6.77 (3.87–11.85)	<0.0001	0.97 (0.65–1.46)	0.90	2.11 (1.56–2.87)	<0.0001
Hip symptoms^c^	0.77 (0.47–1.27)	0.30	2.92 (1.93–4.41)	<0.0001	1.61 (1.17–2.22)	0.004
*AUC (95 % CI):*	0.84 (0.81–0.88)		0.69 (0.64–0.75)		0.71 (0.68–0.75)	
*R* ^*2*^ *: Cragg-Uhler (Nagelkerke):*	0.22		0.08		0.10	

^a^Participants with bilateral TKRs (*n* = 37) at initial visit were excluded from analysis

^b^Participants with bilateral THRs (*n* = 39) at initial visit were excluded from analysis

^c^At initial visit

**Fig. 1:**
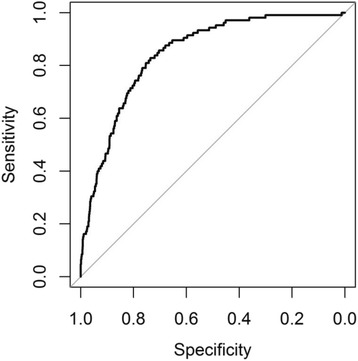
The total predictive power for the incidence of TKR showing the high degree of sensitivity and specificity of a model based on the binary logistic regression analysis in Table 5. AUC 0.84 (0.81–0.88). R^2^: Cragg-Uhler (Nagelkerke): 0.22

In the incidence analysis we added information obtained at the first AGES Reykjavik visit, notably knee and/or hip symptoms and history of previous total joint replacements. These analyses gave sharply contrasting results compared with the late-life prevalence results. Reported knee symptoms were clearly the strongest risk factor followed by BMI in the TKR group. Interestingly, prior total joint replacement operations emerged as a risk factor for the contralateral joints. Individuals with unilateral TKR or THR had an approximately five-fold of having a TJR in the contralateral joint during follow up. Female gender, hand osteoarthritis severity and bone mineral density that were significantly associated with prevalence did not emerge as risk factors. Age showed a modest negative association. The total discriminatory power for TKR was high with an AUC of 0.84 (0.81–0.88) indicating high sensitivity and specificity in predicting TKR (Fig. 1). The only identifiable risk factors for THR were symptoms at first visit and prior TJR.

## Discussion

In this population based study of 5170 elderly individuals, late life prevalence of TKRs showed strong positive associations with BMI and hand OA severity and associations were also detected for finger length ratio and BMD of the spine. Weaker associations were seen for THRs in general but hand OA severity, female gender, BMI and BMD were all significantly positively associated. Incidence analysis during a five year follow-up revealed a very different pattern with symptoms, BMI and prior TKR operations in the contralateral joints emerging as highly significant risk factors for TKRs. Together, these risk factors had a high discriminatory power for incident TKR with an AUC of 0.84. The strongest risk factor for THR was having had a previous THR on the other side.

These results illustrate the importance of separating incidence and prevalence analyses in osteoarthritis. In the clinical situation the incidence results are clearly more relevant, showing a very high predictive power for TKRs and indicating that in the elderly, symptomatic, overweight patients with previous TKR in the contralateral knee are at particular risk for needing TKR operations. Late-life prevalence gives a different kind of information, more relevant to the long term natural course, to the burden and causes of OA. The association with BMI is well known and comparable with that observed by Lohmander and associates [6]. We have previously described the strong association between the presence of severe hand OA and TKR [16], but hand OA was not associated with TKR or THR incidence during follow-up. The prevalence prediction AUC for TKR of 0.71 is quite high, but still leaves room for identification of other factors of importance in the development of knee OA.

Obviously, a predictive model that could assess the burden of OA and the future need for TJRs would be a valuable tool with regard to intervention measures in large joint OA and the planning of resources to meet that need. Several studies have addressed factors associated with the risk of having TJRs [13, 14], but so far the predictive value has been minor, often due to lack of relevant variables and soft endpoints. Recently, an attempt was made to construct a model for the incidence of knee osteoarthritis. Gender, age and BMI were identified as risk factors, but genetics and biomarkers seemed to add little information to the model while radiographic changes did [15]. The current data may be considered as an attempt to construct such a model. The high predictive power for incident TKRs in the present study suggests that this is possible. Regarding the late life prevalence TKR group there are obviously some major factors missing from the current data; previous trauma and minor operations which we do not have data on, and genetics, which despite strong heritability associations, have only contributed with fairly minor risk associations so far [25, 26]. It is likely that, the identification of those likely to need TKRs during their lifetime can also be improved with the use of biomarkers and improved imaging information. Regarding incidence prediction, imaging can obviously contribute significantly to the current predictive model and probably also biomarkers and genetics. With respect to the THRs there appears to be a long way to go towards a predictive model.

Most of the risk factors in the current model have been described previously, but the strength of prior TJR operations as a risk factor was unexpected and constitutes a novel observation in a population based study. In a previous article [17] we had observed a positive association between hand OA, TKRs and THRs indicating the presence of a generalized susceptibility to OA, increasing the likelihood that individuals with end stage OA in one joint would develop the same in another joint, but that study was not done by prospective analysis. Willingness to undergo additional TJRs is subjective in part, and the current data probably indicate satisfaction with the results from previous operation(s), as well as indicating the presence of polyarticular OA subsets. A few authors have addressed this relationship. McMahon found a high frequency of contralateral TKR operations related to radiographic changes [27], and Gillam has reported which joints are most at risk for a second TJR depending on the site of the first operation [28]. Several others have reported polyarticular associations [29] and symmetrical knee OA [30].

We found no evidence of an inverse association between smoking and socioeconomic status and risk for THRs as reported by Mnatzaganian and co-workers in an Australian cohort. [14, 18] The Scandinavian countries have a socialized health care system with equal access for those in need for TJR. Operative indications and prevalence of TJRs are also similar. The absence of associations with education and occupation [18] seems to indicate that there are little or no access barriers to TJRs in Reykjavik and supports the use of TJRs as an endpoint for severe OA. In Iceland and Scandinavia as well as other countries with similar access to health care, TJRs seem to be a suitable endpoint for severe OA whereas standardized criteria equivalent to the indications for TJR could be more suitable in other areas [10].

Possible caveats of this study include the lack of information about incident TJRs in those who did not participitate in the five-year follow-up. The non-participants were on average five years older than the participants and more than 500 of them died during the interval, This limits any conclusions regarding population incidence based on these data. Study participants may represent relatively healthy individuals in the population, and severe cases with early aggressive OA or history of operations with complications may be underrepresented. In addition, some misclassification of exposure regarding occupation and recall bias on physical activity cannot be ruled out. It is also possible that indications for TJR are different late in life compared with other age categories. At this age, there is an increased likelihood of other diseases making the individual unsuitable for TJR operations or at least affecting the decision to operate. This is supported by the finding of a slight negative association with age in the incidence group. Furthermore, both patients and surgeons may be more positive towards TJRs in those with successful operations in the past.

## Conclusions

The results from this large population-based study of the elderly emphasize the differences between factors affecting late life prevalence and risk factors affecting incidence of TJRs. The current data suggest that predictive models for both incidence and late life prevalence of TKRs might be within reach, particularly with the help of additional information from genetics, biomarkers and imaging.

## Abbreviations

OA: osteoarthritis
BMI: body mass index
BMD: bone mineral density
Hs-CRP: high sensitivity C-reactive protein
TJR: total joint replacement
TKR: total knee replacement
THR: total hip replacement
CT: computerized tomography
QCT: quantitative computerized tomography
ROC: receiver operating characteristics
AUC: area under curve
